# Lung Ultrasound in Neonates: A Narrative Review Along With Diagnostic Insights and Early Postnatal Applications

**DOI:** 10.7759/cureus.70487

**Published:** 2024-09-30

**Authors:** Salvatore Michele Carnazzo, Stergios Nasikas, Francesco F Comisi

**Affiliations:** 1 Pediatric Medicine, University of Catania, Catania, ITA; 2 Neonatology, Armand Trousseau Hospital, Sorbonne University, Paris, FRA; 3 Pediatrics, Ospedale Microcitemico, Cagliari, ITA

**Keywords:** lung consolidation, meconium aspiration syndrome, neonatal lung ultrasound, pneumothorax, respiratory distress syndrome, transient tachypnea of the newborn

## Abstract

Ultrasound (US) has become a useful, bedside, noninvasive imaging technique in diagnosing pulmonary diseases, particularly in Neonatology, due to its non-invasive nature, lack of ionizing radiation, and high-quality imaging. This study explores current methodologies, applications, and benefits of thoracic US in neonatal care, highlighting its application in assessing pleural morphology, pulmonary consolidations, and diaphragmatic function. Key ultrasound findings, such as A-lines, B-lines, and the pleural sliding sign, are instrumental in diagnosing various lung conditions, including pneumothorax and respiratory distress syndrome. The present review emphasizes the growing importance of lung ultrasound (LUS) in predicting neonatal intensive care needs, reducing reliance on X-rays, and improving the early diagnosis of conditions like transient tachypnea of the newborn. The use of ultrasound scoring systems enhances diagnostic accuracy, making thoracic ultrasound a valuable addition to neonatal care protocols for real-time, radiation-free assessment from birth.

## Introduction and background

The use of ultrasound in pulmonary disease diagnostics is relatively recent. In the 1960s, the first observations of thoracic ultrasound findings, such as those described by J. Hirsh in pulmonary embolism and pulmonary consolidations, began to emerge [1]. Ultrasound technology was adapted from veterinary medicine, where the detection of pleural line interruption in horses with pneumothorax enabled the description of a characteristic dynamic sign - pleural sliding, whose interruption could be directly attributed to a specific condition, namely, pneumothorax (PNX) [2]. Over the past two decades, several studies and observations have increasingly defined the role of ultrasound in pulmonary pathology, necessitating the development of a universal terminological glossary (e.g., A-lines, B-lines, pleural sliding, curtain sign) to describe ultrasound findings in thoracic examinations. In Pediatrics, due to its relatively easy accessibility, absence of ionizing radiation, reproducibility at the bedside, and excellent image quality granted by the anatomical peculiarities of the child’s thoracic walls, thoracic ultrasound has become widely adopted. In neonatology, the use of thoracic US to identify specific pulmonary conditions from the earliest moments of life may be significant. The ultrasound probe, in a forward-looking manner, may complement current neonatal care equipment and diagnostic tools in the neonatal unit.

Objectives

Our narrative review focuses on the state-of-the-art indications of lung ultrasonography in newborns to help neonatologists differentiate among pathologies and decide on adequate treatment.

Methods

A literature search was performed using PubMed, Scopus, and Google Scholar. Our search was focused on the various techniques and experiences of pulmonary ultrasound imaging in newborns, using observational studies and reviews. Articles were selected and narratively reviewed by two reviewers. In the initial article selection process, a third reviewer was included in times of incongruity between the two primary reviewers.

## Review

Methodological aspects of thoracic ultrasound

**Figure 1:**
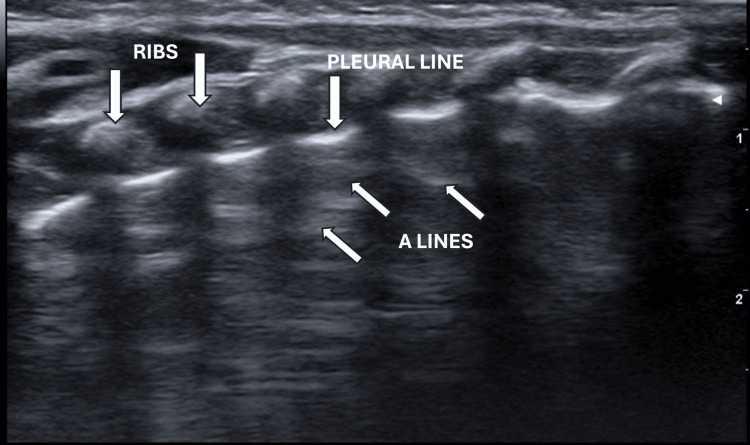
Normal neonatal lung

Despite the widespread use of thoracic ultrasounds, there are no definitive guidelines recommending the use of a specific probe for pulmonary ultrasound evaluation; thus, the probe choice is often empirical. Various types of ultrasound probes are effectively used to study the parietal planes of the chest, characterized by reduced thickness (2-4 cm). Linear probes (7.5-18 MHz) provide high resolution and low penetration, allowing for detailed visualization of pleural morphology. They are particularly useful to examine the superficial planes of the chest. Convex probes (3.5-5 MHz) with medium frequency and resolution are suitable for exploring pulmonary consolidations, pleural effusions, and visualizing the diaphragm. Sector probes (2-3.5 MHz) are primarily used for cardiac studies but may also be employed for general thoracic evaluations in specific cases. The ultrasound image is a two-dimensional reconstruction of anatomical planes in which tissues and organs are visualized in real-time as shades of grey based on their interaction with the ultrasonic wave. However, a pulmonary ultrasound is an exception, as its semiotics rely more on a rich component of artifacts than on anatomical data. In particular, the complete reflection of echoes from the pleural plane generates a hyperechoic linear image known as the pleural line. In a well-aerated lung, this line appears regular and moves horizontally during respiratory acts, producing the sign of "pleural sliding." Lung parenchyma is represented on ultrasound by the echoes of the beam incident on the pleural line, arranged horizontally with spacing corresponding to the distance between the transducer and the pleural plane, referred to as A-lines, horizontal hyperechoic lines parallel to the pleural line of which they are repetitions (Figure 1).

**Figure 2:**
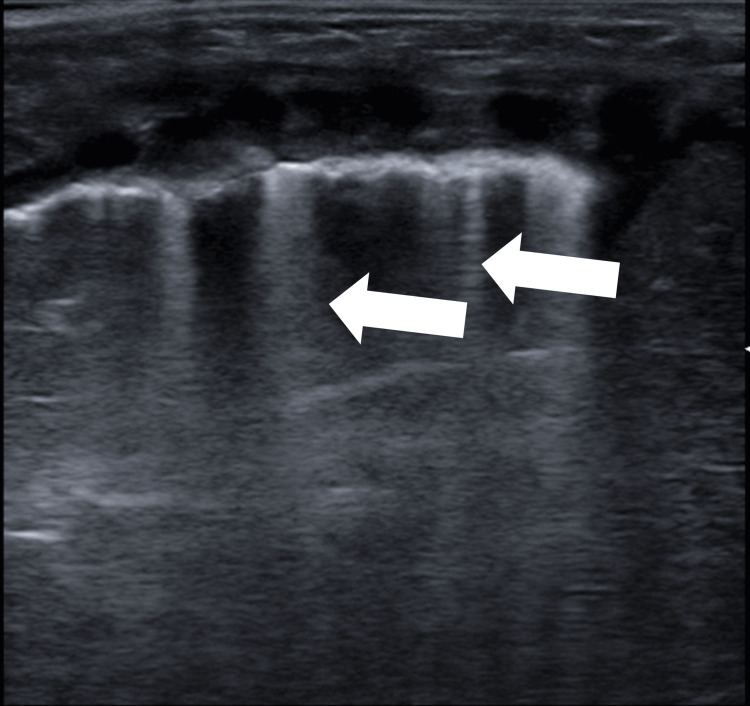
B-lines (arrows) in a newborn

Other semiological elements indicative of partial loss of aeration or increased density of the peripheral lung parenchyma are B-lines, vertical hyperechoic artifacts resembling a “comet tail” arising from the pleural line, extending deep in the parenchyma, following the movement of pleural sliding and erasing A-lines (Figure 2); the coalescence of B-lines results in the image defined as “white lung.” The topographical anatomy of the lung involves dividing the chest into anterior, lateral, and posterior segments. The space between the parasternal line and the anterior axillary line explores the anterior portion of the lung, the one between the anterior and posterior axillary lines explores the lateral portion, and the space posterior to the posterior axillary line explores the posterior portion of the lung. Ultrasound scans are performed by orienting the probe both longitudinally and transversely, exploring all lung portions.

State of the art

A lung ultrasound (LUS) has rapidly gained importance as a diagnostic tool in neonatal care both in the delivery room and in the early hours of life due to its ability to provide real-time images of the lung without the use of ionizing radiation. The clinical application of a LUS has been extensively explored and has proven promising as a potential standard of care. A study by Poerio et al. highlighted how LUS characteristics can predict admission to the neonatal intensive care unit (NICU) in newborns with transient tachypnea of the newborn (TTN) or respiratory distress syndrome (RDS) delivered via cesarean section [3]. This study revealed a significant correlation between ultrasound characteristics and the need for NICU admission, qualifying it as an effective triage tool for such patients. Similarly, research by Badurdeen et al. explored the use of an LUS during neonatal resuscitation to predict the need for surfactant therapy in preterm infants [4]. The results demonstrated that ultrasound provides timely and useful information about lung function, facilitating the rapid identification of infants requiring specific therapeutic interventions. Another significant contribution comes from the study by Blank et al., which evaluated the use of ultrasound in postnatal respiratory transition in term and preterm infants immediately after birth [5]. This study highlighted how an LUS can effectively monitor the postnatal respiratory adaptation phase, providing valuable diagnostic information without interrupting neonatal care in the delivery room. Additionally, Hedstrom et al. demonstrated the added value of combining the LUS with the Silverman respiratory distress score in detecting respiratory distress syndrome in the delivery room [6]. Their findings confirmed that the LUS can complement and enhance early diagnosis of RDS, effectively integrating it into clinical evaluation. Finally, Bhatia et al. emphasized that an LUS, being a non-invasive technique, represents a valuable alternative to traditional methods for diagnosing respiratory distress syndrome [7]. This study highlighted the benefits of the LUS in reducing the need for X-rays and consequently minimizing radiation exposure in neonates. In conclusion, the LUS emerges as a promising and versatile technology for assessing and managing respiratory conditions in neonates in both the delivery room and the early hours of life. Ongoing research in this field could further solidify the role of the LUS as a standard of care in neonatal contexts.

Physiological LUS findings in neonates 

Just minutes after birth in neonates, as observed in the aerated lung tissue of children and adults, it is possible to identify the presence of physiological horizontal artifacts (A-lines), reflections of the ultrasound beam on the pleural line, and B-lines, pathognomonic for interstitial syndrome in adults but considered normal findings in newborns. These findings, observed in both spontaneously delivered and cesarean-born neonates, are associated with the presence of fluid in the fetal lung [8]. A higher number of B-lines can be detected in neonates delivered via cesarean section, presumably due to the absence of thoracic compression during passage through the birth canal [9]. Recent evidence suggests a temporal gradient in ultrasound findings, indicating the presence of coalescent B-lines within the first 10 minutes after birth, sometimes with manifestations of "white lung" until complete pulmonary clearance, with the disappearance of vertical artifacts approximately 36 hours after birth and the appearance of the normal A-line pattern [10]. Some studies suggest that a failure to reduce the number of B-lines at 24 hours post-birth and in the subsequent weeks (between two and eight weeks post-delivery) is directly related to the development of chronic lung disease, especially in preterm infants [11].

Meconium aspiration syndrome

**Figure 3:**
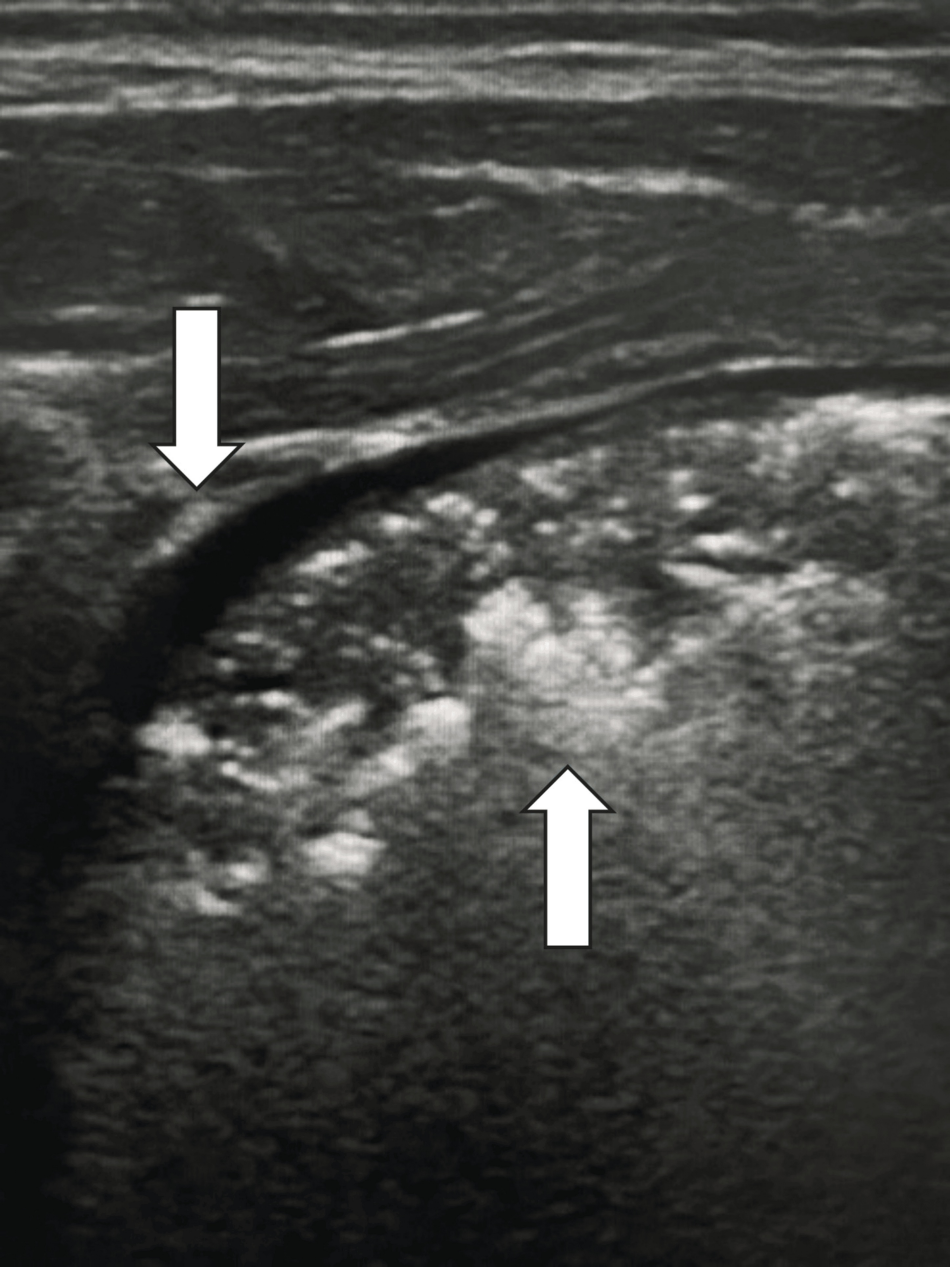
Neonate with meconium aspiration syndrome The lung ultrasound demonstrates a consolidated region in the posterior part of the right lung of a newborn baby, indicated by the lower arrow, 60 minutes after delivery. The ultrasonographic findings consist of loss of the A-line pattern, air bronchograms in the consolidated area, and a pleural effusion between the pleural and the consolidated area, indicated by the upper arrow.

Meconium aspiration syndrome is a frequent cause of neonatal distress, with a higher incidence in term or post-term infants exposed to prenatal stress that leads to sphincter release of the meconium, fetal gasping, and aspiration of meconium-stained amniotic fluid. Meconium in the airways and the resulting chemical pneumonia create an obstruction of the small airways, leading to atelectasis in cases of complete obstruction and air-trapping in cases of partial obstruction. Diagnosis is typically based on anamnesis, clinical findings, and chest radiography [12]. In this context, ultrasound proves to be an accurate, effective, and easy-to-perform examination. The most frequent findings include the presence of pulmonary consolidations with bronchograms, more commonly observed in the right lung (Figure 3). This is likely due to the wider angle and greater inclination of the right main bronchus compared to the left, which promotes more direct flow and thus a more significant accumulation of meconium in the right lung. Other common findings include irregularities of the pleural line, isolated or scattered B-lines (interstitial syndrome), and reduction or disappearance of the normal A-line pattern. Less frequent findings may include atelectasis, absence of lung sliding, pleural effusion, and lung pulse [13]. Ultrasound findings vary with clinical progression; different findings may be observed in the same pulmonary area, reflecting variations in meconium distribution and/or its resolution [14]. Evidence suggests the real-time utility and effectiveness of chest ultrasound in monitoring bronchoalveolar lavage procedures aimed at removing bronchial obstruction caused by the meconium. In the follow-up ultrasound, a return to normal lung aeration can be observed, indicated by the reduction of the interstitial pattern (i.e., B-lines and white lung) and the presence of an A-line pattern [15].

Transient tachypnea of the newborn

Transient tachypnea of the newborn is one of the most common causes of respiratory distress in neonates, typically presenting within the first hour after birth with tachypnea and hypoxemia. Although it is associated with low morbidity and resolves, by definition, within 24-72 hours, diagnosis can be complicated by the low specificity and sensitivity of clinical signs and symptoms [16]. The commonly used diagnostic tool is chest radiography, which reveals peri-hilar streaks attributed to lymphatic congestion and fluid in the fissures. However, it is rare to observe areas of pulmonary infiltrates or small pleural effusions [17]. In such conditions, the use of an ultrasound demonstrates diagnostic accuracy sufficient to make a diagnosis based on the distinctive ultrasound findings [18]. Characteristic findings include pulmonary edema, which presents as the alveolar-interstitial syndrome (sensitivity 94%, specificity 88%), irregularity of the pleural line (sensitivity 100%, specificity 0%), “white lung” in the absence of consolidations, and a pathognomonic sign known as the “double lung point sign” (sensitivity 94%, specificity 100%) [19]. The latter is characterized by different echogenicity between the upper and lower lung fields, indicated by the presence of compact, more confluent, B-lines at the bases compared to fewer or absent B-lines in the upper lung fields [20]. This finding is generally evident in both lungs, though not always symmetrical, with greater frequency in the right lung [21].

Neonatal respiratory distress syndrome

**Figure 4:**
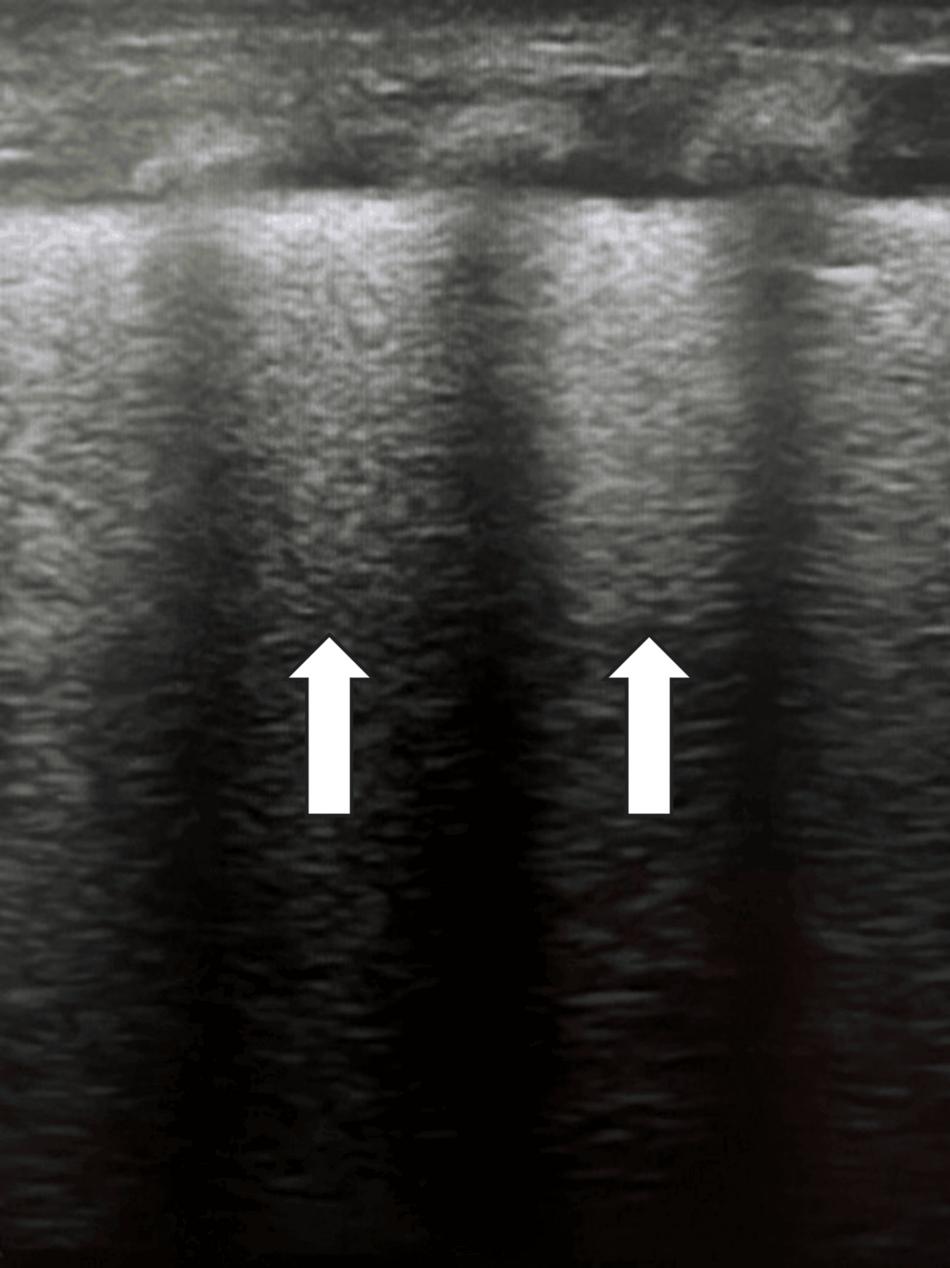
Neonate with hyaline membrane disease The lung ultrasound demonstrates a compact B-line pattern and the absence of an A-line pattern in the anterior superior lung field of a premature newborn of 29 gestational weeks, 30 minutes after the delivery.

Neonatal respiratory distress syndrome is a clinical condition caused by surfactant deficiency, primarily affecting premature infants and leading to reduced pulmonary compliance and functional residual capacity [22]. Radiographically, a bilateral and diffuse pattern of ground-glass opacity with air bronchograms and reduced lung expansion can be observed, although these findings are attenuated by the introduction of surfactant therapy. Radiography can detect complications of RDS such as pneumothorax, pneumomediastinum, pneumopericardium, interstitial emphysema, and pulmonary hemorrhage [23]. In RDS, pulmonary ultrasound shows the presence of pulmonary consolidation with air bronchograms, a pattern of compact B-lines ranging from moderate to severe, irregular and thickened pleural lines, and a reduction in the A-line pattern (Figure 4) [24].

**Figure 5:**
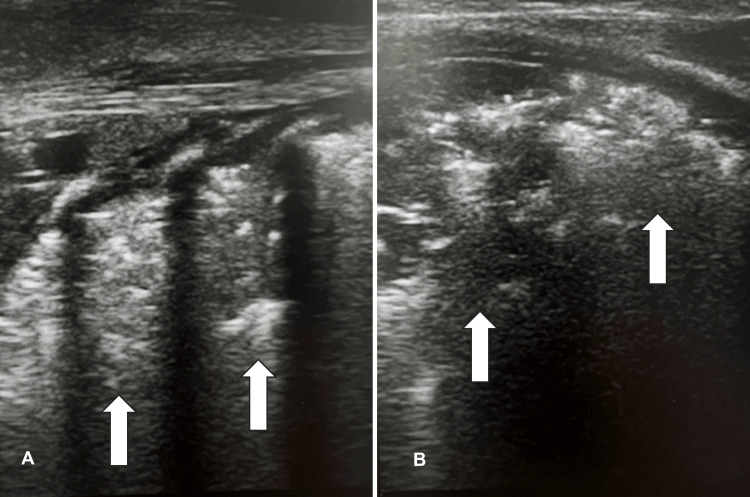
Neonate with Bronchopulonary Dysplasia The lung ultrasound demonstrates extended consolidations with significant air bronchograms and the absence of A- and B-line patterns. A. Lateral view of the consolidated lung. B. Posterior view of the consolidated lung.

Ultrasound scores, such as the Lung Ultrasound Score and the Neonatal Pulmonary Score (NPS), have been evaluated in the literature for their predictive power regarding the need for surfactant therapy. Specifically, the Lung Ultrasound Score quantifies visible changes on pulmonary ultrasound in premature infants, including patterns such as the presence of B-lines, A-lines, pleural line irregularities, and the distribution and severity of abnormalities, providing a higher score relative to the severity of the condition [25]. Similarly, NPS evaluates the distribution and intensity of B-lines, where a higher score can also indicate greater pulmonary impairment and the need for surfactant treatment. In the Lung Ultrasound Score, they have used scoring and patterns - Score 0 indicates A-pattern (defined by the presence of only A-lines, Socre 1 - B-pattern (defined as the presence of 3 B-lines, Score 2, severe B-pattern (defined as the presence of crowded and coalescent B lines with or without consolidations and Score 3, extended consolidation [26]. The presence of diffuse and compact B-lines throughout the lung, both in the apices and bases bilaterally, and in some cases forming a white lung, represents a distinctive ultrasound finding compared to transient tachypnea of the newborn (TTN). In some cases, sub-pleural hypoechoic consolidations of likely atelectatic nature can be observed. Additionally, thoracic ultrasound can detect complications of respiratory distress syndrome sometimes with greater accuracy than chest radiography such as bronchopulmonary dysplasia (Figure 5), pulmonary hemorrhage, pneumothorax, consolidations, atelectasis, micro-abscesses, and microconsolidations [27].

Pneumothorax 

The use of ultrasound in the management of pneumothorax in pediatric patients is well-established [28]. Pneumothorax in neonates is a potentially life-threatening condition and is relatively common as a complication in patients undergoing mechanical ventilation or experiencing air leaks, often associated with respiratory distress syndrome or meconium aspiration. Symptomatic pneumothorax occurs in 0.08% of all live births and in 7% of infants with low birth weight (weight <1500 g), and in many cases, symptoms may not be apparent in 90% of cases [29]. The diagnostic approach for patients with characteristic symptoms includes chest radiography and high-intensity, fiber-optic transillumination; the latter is not always feasible, especially if the air collection is small or located posteriorly or laterally. Thoracic ultrasound has demonstrated excellent sensitivity and specificity in diagnosing pneumothorax, particularly when compared to radiography [30]. The most common findings are the absence of lung sliding, B-lines, and lung pulse (rhythmic movement of the pleura synchronized with the heartbeat), evidence of a lung point (areas of the pleural line where a sudden interruption of pleural sliding is observed) in B-mode [31]. Ultrasound study of a healthy lung in M-mode reveals the typical ‘seashore sign’. In cases of pneumothorax, a ‘barcode or stratosphere sign’ is observed due to the presence of air between the visceral and parietal pleura, which abolishes lung sliding [32]. Cattarossi et al. published a study showing that in a cohort of 33 neonates with pneumothorax, pulmonary ultrasound had superior accuracy for diagnosing pneumothorax, particularly in symptomatic patients using chest radiography and transillumination as reference standards [33]. Additionally, the study conducted by Raimondi et al., known as the LUCI (Lung Ultrasound in the Crashing Infant) protocol, demonstrated that pulmonary ultrasound is a highly effective tool for diagnosing pneumothorax in critically ill neonates [34]. This study, which involved a large group of neonates, confirmed that ultrasound provides superior diagnostic accuracy compared to radiography and transillumination, thus establishing a new benchmark in the management of pneumothorax in neonates.

Recommendations

Notwithstanding a few limitations of the technique as described by some authors [35], the routine incorporation of an LUS into neonatal care is highly recommended. LUS should be established as a frontline diagnostic tool in neonatal intensive care units, due to its ability to provide real-time, detailed imaging without the risks associated with ionizing radiation. Its application plays a key role in the early diagnosis and management of common neonatal respiratory conditions such as TTN, RDS, and pneumothorax. Standardized training programs for neonatologists and NICU staff should be implemented to ensure proficiency in the use of LUS. Furthermore, incorporating LUS into clinical guidelines and care protocols will facilitate its consistent and effective use, improving neonatal outcomes.

Future directions

Future research should focus on the development of comprehensive ultrasound scoring systems specifically tailored for newborns. Studies should also explore the long-term outcomes of neonates diagnosed and managed using LUS to establish its efficacy in reducing chronic respiratory conditions. Furthermore, technological advancements could enhance the accessibility of LUS in different clinical settings, including resource-limited environments. A multidisciplinary approach between neonatologists, radiologists, and engineers will be essential to drive these innovations, aiming to establish LUS as a universal standard of care in neonatal respiratory management

## Conclusions

Pulmonary ultrasound is a valuable and effective method in the diagnosis and management of neonatal respiratory issues. Its ability to provide quick, real-time images without the risks associated with radiation makes it an ideal tool in neonatal care. By detecting specific patterns, such as A-lines and B-lines, and observing the movement of the pleura and lung consolidations, LUS provides key information about a newborn's lung health. The findings discussed in this review highlight that LUS is not only accurate but often more reliable than traditional X-rays to diagnose conditions like transient tachypnea of the newborn, respiratory distress syndrome, and pneumothorax. LUS also enhances clinical decision-making, helping ensure timely interventions, which could potentially shorten NICU stays and reduce the intensity of required treatments. Its role in guiding the use of therapies like surfactant administration shows its practical value in the clinical setting. Moreover, by reducing the need for repeated X-rays, LUS minimizes radiation exposure, which is a significant concern for neonates. For LUS to be used effectively, there must be standardized protocols and comprehensive training for healthcare providers. This will help maximize its diagnostic potential. Ongoing research should focus on developing more refined ultrasound scoring systems that can predict long-term outcomes and incorporate these into everyday clinical practice. Advances in technology, making ultrasound devices more portable and easier to use, will also expand the reach of LUS, especially in settings with limited resources. In summary, LUS has established itself as a vital tool in neonatal care. It offers a safe, efficient, and effective way to diagnose and manage respiratory conditions from the very first moments of a newborn's life. By incorporating LUS into standard practice, we can improve care quality and outcomes for our most vulnerable patients.
